# Acute stress transiently activates macrophages and chemokines in cervical lymph nodes

**DOI:** 10.1007/s12026-023-09409-w

**Published:** 2024-02-14

**Authors:** Akihiro Dohi, Tadahide Noguchi, Masako Yamashita, Kenichi Sasaguri, Toshiharu Yamamoto, Yoshiyuki Mori

**Affiliations:** 1 Department of Dentistry, Oral and Maxillofacial Surgery, Jichi Medical University, Tochigi, 329-0498 Japan; 2 Brain Functions and Neuroscience Division, Department of Oral Science, Graduate School of Dentistry, Kanagawa Dental University, Yokosuka, Kanagawa Japan

**Keywords:** CXCL1, CXCL2, CXCR2, Stress response, Macrophage, Lymph node

## Abstract

Acute restraint stress (RS) is routinely used to study the effects of psychological and/or physiological stress. We evaluated the impact of RS on cervical lymph nodes in rats at molecular and cellular levels. Male Sprague–Dawley rats were subjected to stress by immobilization for 30, 60, and 120 min (RS30, RS60, and RS120, respectively) and compared with rats of a no-stress control (C) group. The expression of genes encoding chemokines CXCL1/CXCL2 (*Cxcl1* and *Cxcl2*) and their receptor CXCR2 (*Cxcr2*) was analyzed using reverse transcription-quantitative PCR (RT-qPCR) and microarray analyses. Immunohistochemistry and in situ hybridization were performed to determine the expression of these proteins and the macrophage biomarker CD68. Microarray analysis revealed that the expression of 514 and 496 genes was upregulated and downregulated, respectively, in the RS30 group. Compared with the C group, the RS30 group exhibited a 23.0-, 13.0-, and 1.6-fold increase in *Cxcl1*, *Cxcl2*, and *Cxcr2* expression. Gene Ontology analysis revealed the involvement of these three upregulated genes in the cytokine network, inflammation, and leukocyte chemotaxis and migration. RT-qPCR analysis indicated that the mRNA levels of *Cxcl1* and *Cxcl2* were significantly increased in the RS30 group but were reverted to normal levels in the RS60 and RS120 groups. *Cxcr2* mRNA level was significantly increased in the RS30 and RS120 groups compared with that in the C group. RS-induced CXCL1-immunopositive cells corresponded to B/plasma cells, whereas CXCL2-immunopositive cells corresponded to endothelial cells of the high endothelial venules. Stress-induced CXCR2-immunopositive cells corresponded to macrophages. Psychological and/or physiological stress induces an acute stress response and formation of an immunoreactive microenvironment in cervical lymph nodes, with the CXCL1/CXCL2–CXCR2 axis being pivotal in the acute stress response.

## Introduction

Lymph nodes are secondary lymphoid organs that play a vital role in regulating immune responses in animals and initiating primary immune responses in other secondary lymphoid organs [1, 2]. A lymph node comprises the cortex, paracortex, and medulla, along with the adjoining areas encompassing antigen-presenting cells, antigenic substances from the lymph, and lymphocytes recruited into the lymph nodes from the blood [2–4]. The lymphatic vessel system allows the passage of signals from local pre-inflammatory and inflammatory sites [5]. The cervical lymph nodes are crucial in the regulation of immune responses in the maxillofacial area [6]. In humans, the cervical lymph nodes are involved in the pathway of metastases from maxillofacial carcinoma and are regarded as sentinel lymph nodes of the carcinoma in such areas. Understanding the basic nature of cervical lymph nodes would provide important insights into the mechanisms of metastases through cervical lymph nodes, even in animal models.

Chemokines are a superfamily of structurally similar proteins, comprising small cytokines that chemotactically recruit cells and mediate angiogenesis, immune response, homeostasis, and stem cell trafficking [7]. Functionally, chemokines are divided into three categories, namely homeostatic, pro-inflammatory, and dual-function [7], whereas structurally, they are classified into four families, namely CXC, CC, CX3C, and C [8]. Accordingly, their receptors are denoted as CXCR, CCR, CX3CR, and CR, respectively [9]. Of these, CXC chemokines are further subdivided into two groups: those with an ELR (Glu-Leu-Arg) motif preceding the first Cys and those without this motif [9]. Chemokines with an ELR, including CXCL1–3, 5–8, and 15, activate and direct neutrophils and exert angiogenic roles. Those without an ELR, including CXCL4, 9–11, and 14, act mainly on monocytes and lymphocytes and exert angiostatic effects [9]. CXCL1 (also known as GROα or MGSA-α) and CXCL2 (GROβ or MGSA-β) belong to the ELR group and share a common receptor, CXCR2 [10].

Stress induces changes in several cellular and humoral immune parameters. For example, immunological performance is affected by events such as immobilization, forced swimming, and electric shock [11–13]. Chronic stress suppresses immunity, whereas acute stress can enhance immunity [14, 15]. We have previously clarified the effects of acute stress on various brain regions [16–18]. The brain is an essential organ that protects the body from stress in concert with the immune system [19–21]; however, the effects of acute stress on lymphatic organs are not fully understood. Therefore, in this study, we aimed to elucidate the effects of acute stress on the cervical lymph nodes, a representative secondary lymphatic organ, in an experimental in vivo model. We examined changes in the expression levels and immunochemical characteristics of macrophages, erythrocytes, CXCL1, CXCL2, and CXCR2 in rat models.

## Materials and methods

### Study animals

Forty male Sprague–Dawley rats (10–12 weeks old, body weight 432 ± 30 g; SLC Japan, Hamamatsu, Japan) were used in this study. Experiments were conducted from December 2019 to August 2021. All animals were housed in an experimental animal facility, where the managers monitored animal health and behavior twice per day. The animals were maintained in a temperature-controlled room (23 ± 3 °C) with a 12/12 h light/dark cycle (lights were switched on at 7:00 h). Two to three rats were housed per cage (made of polypropylene; dimensions: 307 mm wide, 405 mm deep, and 227 mm high), and the rats had free access to water and food. The animals were treated humanely, taking all due measures to alleviate suffering. The Review/Ethics Committee of Jichi Medical University approved all experimental procedures, which were conducted according to the University Guidelines for Animal Experimentation and National Institutes of Health Guide for the Care and Use of Laboratory Animals (December 2019 Approval number: 18038-03).

### Stress induction

The animals were divided randomly into four groups (*n* = 6): a control group (C) and three experimental groups subjected to restraint stress (RS) for 30 min (RS30), 60 min (RS60), and 120 min (RS120). Animals were tied to a wooden board for 30, 60, and 120 min, respectively, to induce RS. Leg fasteners held the rats in the supine position (spread-eagle position). All stress induction procedures and euthanasia were performed between 11:00 and 17:00 h. RS was induced as described in our previous reports [17, 18, 22].

### Sample preparation

Immediately after each stress induction time was over, the animals were transferred into an airtight plastic case, anesthetized using inhalational anesthesia with 5% halothane (2-bromo-2-chloro-1,1,1-trifluoroethane; Takeda Chemical Industries, Osaka, Japan), and euthanized by intraperitoneal overdose of sodium pentobarbital (85 mg/kg; FUJIFILM Wako Chemical Corp., Osaka, Japan). The death of animals was confirmed by checking for whole body atony and respiratory arrest. After decapitation, the cervical lymph nodes were harvested from each group for microarray and reverse transcription-quantitative PCR (RT-qPCR) analyses. The organs were collected into plastic tubes and stored at −80 °C until analysis.

### RNA isolation

The total RNA was extracted from the cervical lymph nodes of the four groups using ISOGEN reagent (Nippon Gene Co. Ltd., Toyama, Japan), according to the manufacturer’s instructions. The total RNA concentration and purity were measured spectrophotometrically using a NanoDrop1000 (NanoDrop Technologies, Wilmington, DE, USA). RNA integrity was monitored by electrophoresis on an Agilent Bioanalyzer RNA 6000 Nano (Agilent Technologies, Santa Clara, CA, USA).

### Microarray analysis

The extracted RNA (100 ng) was subjected to Cy3-labeled cRNA synthesis using a Low Input Quick Amp Labeling Kit (5190-2305: Agilent Technologies). The amplified RNA and dye incorporation were quantified using the Agilent Bioanalyzer RNA 6000 Nano and hybridized to a microarray chip (SurePrint G3 Rat GE Microarray 8X60K ver. 2.0, Agilent Technologies). After hybridization, the chips were washed using the Gene Expression Wash Pack. The arrays were scanned with an Agilent SureScan G4900DA, and fluorescence intensity was extracted using Feature Extraction software ver. 11.5.1.1.

Raw data of the microarray intensities were normalized using the 75th percentile shift method with GeneSpring software ver. 14.9 (Agilent Technologies) for inter-microarray variability. Differential gene expression was defined as a two-fold change relative to that of the control. We performed Gene Ontology (GO) analysis to summarize the biological functions of the selected genes. The data were then processed using Fisher’s exact test and multiple test correction to identify significant over-representation of GO annotations belonging to the selected genes.

### RT-qPCR

The total RNA was extracted using the ISOGEN reagent (Nippon Gene, #311-07361), as described above, from the four groups. These concentrations were determined using the NanoDrop1000. cDNA was synthesized using a Transcriptor First-Strand cDNA synthesis kit (#04897030001, Roche Diagnostics Ltd., Basel, Switzerland), following the manufacturer’s protocol. Briefly, the template–primer mixture was denatured by heating the tube for 10 min at 65 °C in a heated block. The tube was briefly centrifuged and immediately cooled on ice. Master mix was added, and the tube was placed in a thermal cycler with a heated lid for 60 min at 50 °C. Reverse transcriptase was inactivated by heating the tube to 85 °C for 5 min, and the samples were chilled on ice. The synthesized cDNA stock was stored at −25 °C until analysis. qPCR was performed on a LightCycler® TaqMan Master (#04735536001, Roche Diagnostics Ltd.) using commercially available TaqMan probes with a dye label (FAM) on the 5′ end and a minor groove binder and a non-fluorescent quencher on the 3′ end, CXCL1 (Rn00578225_m1) [23], CXCL2(Rn00586403_m1) [24, 25], CXCR2(Rn02130551_s1) [24, 26], and GAPDH(Rn01775763_g1) [27] (Thermo Fisher Scientific, Waltham, MA, USA). The thermal cycling conditions provided for the LightCycler® 2.0 System were as follows: enzyme activation: 95 °C for 10 min, 45 cycles of amplification: 95 °C for 10 s, 60 °C for 30 s, and signal detection at 72 °C for 1 s, and cooling at 40 °C for 30 s. *Gapdh* was used as the reference gene to normalize the expression levels.

### Immunohistochemical analysis

We performed a histological examination to identify the tissues exhibiting increased *Cxcl1*, *Cxcl2*, and *Cxcr2* mRNA levels in the C and RS30 groups. For immunohistochemistry, animals (*n* = 3 in each group) were anesthetized and euthanized as described above. The animals were then transcardially perfused with 0.85% NaCl followed by 4% formaldehyde and 0.2% picric acid in 0.1 M sodium phosphate buffer (PB; pH 6.9). The cervical lymph nodes were dissected and fixed in the same fixative for 24 h. After immersing the samples in 20% sucrose, the lymph nodes were cut into 10-μm-thick sections using a sliding microtome (Yamato Kohki Industrial Co. Ltd., Asaka, Japan) equipped with a frozen stage. The sections were stored in 0.1 M PB (pH 7.4) containing 0.9% phosphate-buffered saline (PBS) until immunostaining. Some sections were stained with hematoxylin and eosin to examine the general histology. Immunohistochemistry was performed as described in our previous report [22]. Briefly, the sections were washed overnight and incubated with the following primary antibodies: mouse anti-CD68 antibody (ab31630; Abcam, Cambridge, UK; 1:100), rabbit anti-CXCL1 polyclonal antibody (bs-10234R, Bioss Antibodies, Boston, MA, USA; 1:500), rabbit anti-CXCL2 polyclonal antibody (LS-C294372; LifeSpan BioSciences, Inc., Seattle, WA, USA; 1:500), and rabbit anti-CXCR2 polyclonal antibody (orb1720; Biorbyt Ltd., Cambridge, UK; 1:500). After washing, the sections were incubated with biotinylated goat anti-rabbit IgG (BA-1000; Vector Laboratories, Burlingame, CA, USA; 1:100) for 1 h. For CD68, the sections were incubated with biotinylated donkey anti-mouse IgG (Millipore Corporation, Billerica, MA, USA; 1:100). The sections were washed and incubated for 30 min at room temperature with avidin–biotin–horseradish peroxidase complex (PK-6100; Vector Laboratories; 1:200). After the final wash, the sections were reacted with 0.02% 3,3′-deaminobenzidine tetrahydrochloride and 0.005% hydrogen peroxide in 0.05 M Tris-HCl (pH 7.4), counterstained with thionine, and coverslipped using Malinol (Muto Pure Chemicals Co. Ltd., Tokyo, Japan). For double staining of CD68 and CXCR2, the samples were treated with Alexa Fluor 488-conjugated donkey anti-mouse IgG specific for CD68 (ab150105; Abcam; 1:100) and Texas red–conjugated donkey anti-rabbit IgG specific for CXCR2 (GTX26800; GeneTex, Inc., Irvine, CA, USA; 1:100). PBS was used for all washing steps, and PBS containing 1% bovine serum albumin and 0.3% Triton X was used to dilute the antibodies. The cells were counted using printed images, and the areas with cells were calculated from the weights of printed papers.

In situ hybridization (ISH) was performed according to the manufacturer’s protocol (GenoStaff Co., Ltd., Tokyo, Japan). Animals (*n* = 3 in each group) were sacrificed as described above, except that 4% formaldehyde in PB (pH 7.4) was used as the fixative. The cervical lymph nodes were collected and embedded in paraffin wax after dehydration. Next, 6-μm-thick sections were mounted on MAS-coated glass slides (Matsunami Glass Ind., Ltd., Osaka, Japan). The sections were dewaxed using xylene and then hydrated in decreasing ethanol concentrations followed by distilled water. The sections were treated with 0.2% hydrochloric acid and 5 μg/mL protease K (FUJIFILM Wako Chemical Corp.) at 37 °C for 10 min each. After washing with PBS and hybridization buffer, the sections were hybridized to relevant probes at 60 °C overnight. Digoxigenin-labeled antisense and sense RNA probes were synthesized by GenoStaff Co., Ltd. (for rat CXCL1, sequence position: 636–864, size: 229 bases; for rat CXCL2, sequence position: 727–1047, size: 321 bases; for rat CXCR2, sequence position: 16–266, size: 251 bases). After washing with hybridization buffer, the sections were washed with 50% formamide for 10 min at 60 °C. The sections were washed with hybridization buffer and then with Tris-HCl buffer (pH 7.0) containing 0.8% NaCl, 0.02% KCl, and 0.2% Tween-20 at room temperature. Digoxigenin was detected with alkaline phosphatase-labeled anti-digoxigenin Fab fragments (32871920; Roche Diagnostics, Mannheim, Germany; 1:1,000 dilution), and signals were visualized using a mixture of nitrotetrazolium blue chloride (Sigma–Aldrich, St. Louis, MO, USA) and 5-bromo-4-chloro-3-indolyl phosphate *p*-toluidine salt (Sigma–Aldrich) in Tris-HCl buffer (pH 9.5). The sections were counterstained with Kernechtrot staining solution (Muto Pure Chemicals Co., Ltd.) and coverslipped with Malinol.

### Statistical analysis

Data are presented as the mean ± standard error of the mean. Differences between groups were analyzed by one-way analysis of variance, followed by Tukey’s post-hoc test. Results with *P* < 0.05 were considered statistically significant. Immunohistochemical and in situ histochemical data were statistically analyzed using *t*-tests. Statistical analysis was performed using Statcel4 software (OMS Publishing, Inc., Saitama, Japan).

## Results

### Screening of differentially expressed genes

**Fig. 1:**
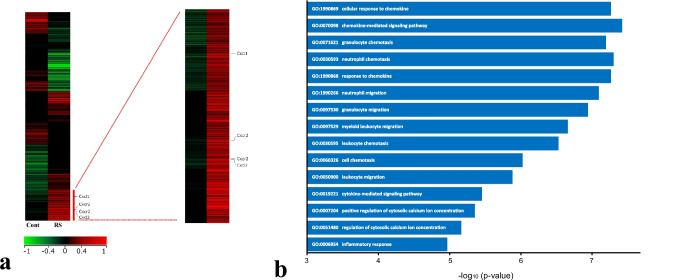
Heat-map clustering analysis and Gene Ontology (GO) functional enrichment analysis. **a** Heat-map representing differentially expressed genes in cervical lymph nodes of control and 30-min restraint stress rats as obtained in microarray analysis. **b** Top 15 GO terms and biological processes related to the three upregulated genes

We screened a total of 26,186 genes using microarray analysis. Among them, the expression of 514 and 496 genes was upregulated and downregulated, respectively, by 2-fold or more in the RS30 group compared with that in the C group. Notably, we observed 23- and 13-fold higher expression levels of *Cxcl1* and *Cxcl2*, respectively, in the RS30 group than in the C group. Furthermore, the expression of *Cxcr2* was upregulated by approximately 1.6-folds in the RS30 group compared with that in the C group (Fig. 1a). Using a database (Gene Ontology Consortium (http://www.geneontology.org)), microarray analysis data were integrated to identify the GO terms and biological processes related to the three upregulated genes. The top 15 significantly enriched GO terms associated with the three upregulated genes are presented in Fig. 1b.

### mRNA expression analysis using RT-qPCR

**Fig. 2:**
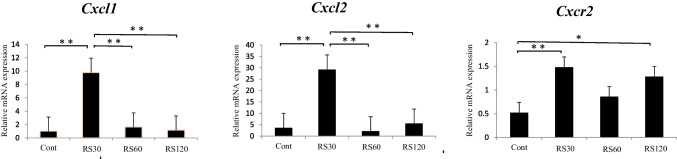
RT–qPCR analysis of *Cxcl1*, *Cxcl2*, and *Cxcr2* mRNA in cervical lymph nodes. Relative mRNA expression of *Cxcl1*, *Cxcl2*, and *Cxcr2* in the four groups: control (Cont), restraint-stressed for 30 min (RS30), 60 min (RS60), and 120 min (RS120) (*n* = 6). **P < 0.01; *P < 0.05 vs. control. Data are presented as the mean ± standard error of the mean

Stress immediately induced transient upregulation of *Cxcl1/2* mRNA expression in the RS30 group (*P* < 0.01 compared with the C group based on a Tukey’s post-hoc test). Notably, the expression levels were significantly downregulated in the RS60 and RS120 groups (*P* < 0.01) compared with the expression levels in the RS30 group, and the levels recovered to the normal levels in the C group. In contrast, the mRNA levels of *Cxcr2* were significantly increased in the RS30 and RS120 groups (*P* < 0.05) compared with that in the C group. The mRNA levels tended to remain at higher levels in the RS60 group than in the C group, although the differences were not significant (Fig. 2).

### General histopathology findings

**Fig. 3:**
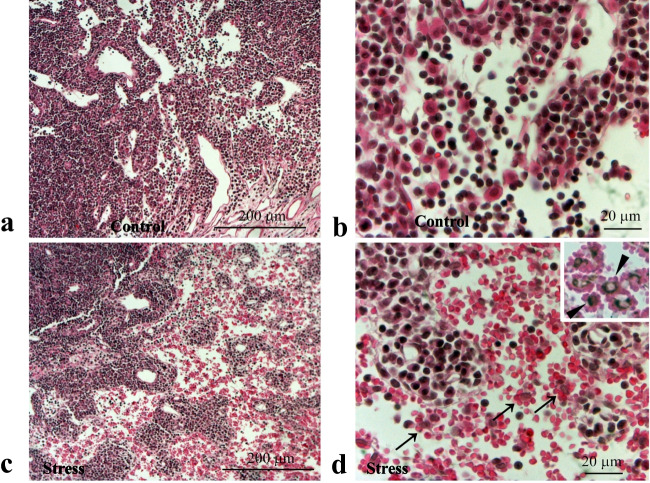
Erythrocyte accumulation in the 30-min restraint-stressed group. Low (**a**, **c**) and high (**b**, **d**) magnification micrographs of hematoxylin and eosin-stained sections of control (**a**, **b**) and 30-min restraint-stressed (**c**, **d**) animals. Arrows in **d** indicate erythrocyte rosette formation in the medullary sinus. Inset in **d** indicates CD68-immunoreactive macrophages at the center of each rosette (arrowhead). Scale bars: **a** and **c** = 200 μm, **b** and **d** = 20 μm

Cervical lymph nodes in the RS30 group showed frequent erythrocyte accumulation in the medullary sinus; this accumulation was not observed in the C group (Fig. 3a, b). The accumulated erythrocytes also formed rosettes enclosing macrophages (Fig. 3c, d). Erythrocyte accumulation was rarely observed in the RS60 group and was not detectable in the RS120 group.

### Immunohistochemical and ISH findings

**Fig. 4:**
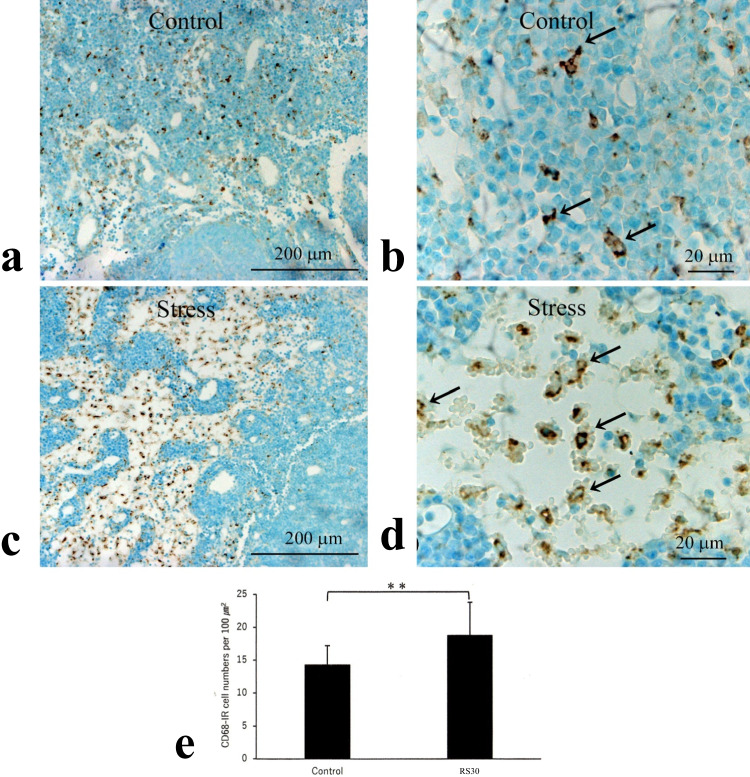
Increases in CD68-IR cells and their cell numbers in the 30-min restraint-stressed group. Low (**a**, **c**) and high (**b**, **d**) magnification micrographs of immunohistochemically stained CD68-immunoreactive (-IR) cells in the medullary sinus of control (**a**, **b**) and 30-min restraint-stressed (**c**, **d**) animals. Arrows in **b** and **d** indicate CD68-IR cells. Scale bars: **a** and **c** = 200 μm, **b** and **d** = 20 μm. **e** Bar graph of the number of CD68-IR cells per 100 μm^2^ in control and 30-min restraint-stressed (RS30) animals. **P < 0.01 vs. control

CD68-immunoreactive macrophages were mainly localized in the medullary sinus (Fig. 4a-e). In the cervical lymph nodes of the C group, CD68-immunoreactive macrophages were slender and scattered (Fig. 4a, b). In contrast, the macrophages in the RS30 group were swollen and more densely located in the lymph nodes (Fig. 4c, d). The number of CD68-immunoreactive cells (per 100 μm^2^) in the medullary sinus was 14.3 ± 2.9 and 18.8 ± 5.0 in the C and RS30 groups, respectively (*P* < 0.01) (Fig. 4e).

**Fig. 5:**
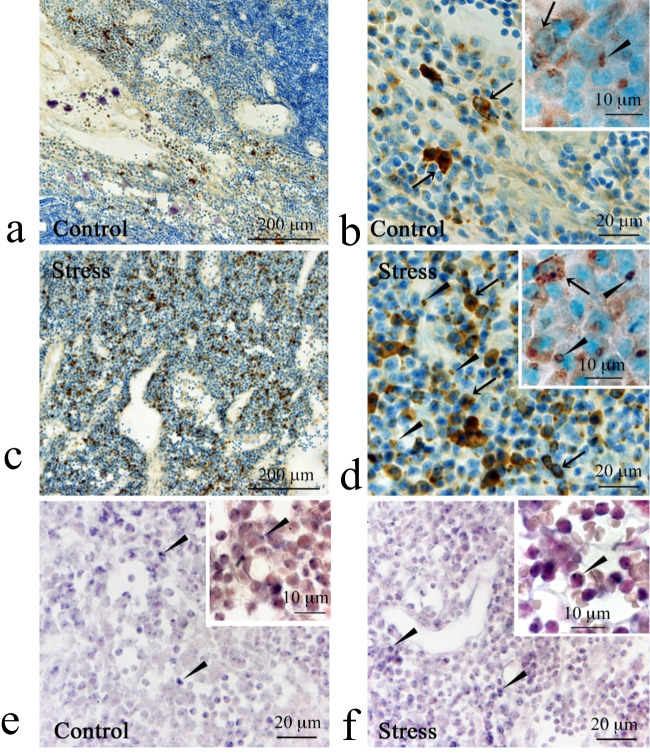
Increases in CXCL1-IR and *Cxcl1*-ISH-positive cells in the 30-min restraint-stressed group. Low (**a**, **c**), medium (**b**, **d**), and high (insets) magnification micrographs of CXCL1-immunoreactive (-IR) cells and puncta in the medullary cords of the control (**a**, **b**) and 30-min restraint-stressed (**c**, **d**) animals. Medium (**e**, **f**) and high (insets) magnification micrographs of in situ hybridization (ISH) histochemical signals of *Cxcl1* in the medullary cords of the control (**e**) and 30-min restraint-stressed (**f**) animals. Arrows in **b**, **d** and insets indicate CXCL1-IR cells; arrowheads in **b**, **d** and insets indicate CXCL1-immunoreactive puncta. Arrowheads in **e**, **f** and insets indicate ISH histochemical signals from cells in the medullary cords. Scale bars: **a** and **c** = 200 μm; **b** and **d**–**f** = 20 μm; insets = 10 μm

**Fig. 6:**
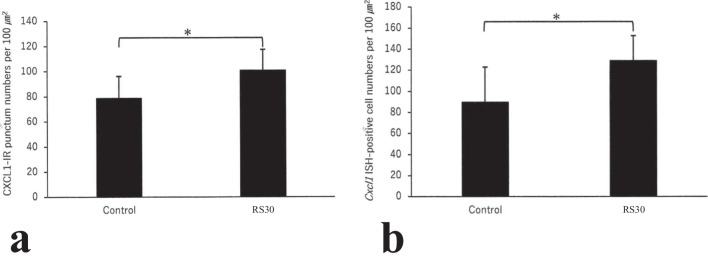
Increases in CXCL1-IR punctum numbers and *Cxcl1*-ISH-positive cell numbers in the 30-min restraint-stressed group. Bar graphs indicating the number of CXCL1-immunoreactive (-IR) puncta (**a**) and *Cxcl1* in situ hybridization (ISH)-positive cells (**b**) in the control and 30-min restraint-stressed (RS30) animals per 100 μm^2^. *P < 0.05 vs. control

CXCL1-immunoreactive cells were mainly localized in the medullary cords where B/plasma cells were present (Fig. 5a-d). Immunoreactive puncta were eccentrically localized to the cell nucleus (Fig. 5b, d), presumably corresponding to cisternae of the rough endoplasmic reticulum and/or Golgi complex of plasma cells. In some presumed B cells, immunoreactivity was extended to the whole cell bodies (Fig. 5b, d). Based on the cell size (7–9 μm in diameter), paucity of the cytoplasm, and location in the B/plasma cell territories, these immunoreactive cells were predicted to be B cells. The number of these cells was smaller than that of immunoreactive puncta-bearing cells. The number of immunoreactive puncta per 100 μm^2^ was 78.8 ± 17.3 and 100.8 ± 16.6 in the C and RS30 groups, respectively (*P* < 0.05) (Fig. 6a). Concomitantly, ISH signals were observed in areas eccentrical to the cell nucleus (Fig. 5e, f). The number of B/plasma cells with these features was larger in the RS30 group (Fig. 5f) than in the C group (Fig. 5e). The number of *Cxcl1* ISH-positive cells per 100 μm^2^ was 89.8 ± 33.2 and 129.2 ± 23.5 in the C and RS30 groups, respectively (*P* < 0.05) (Fig. 6b).

**Fig. 7:**
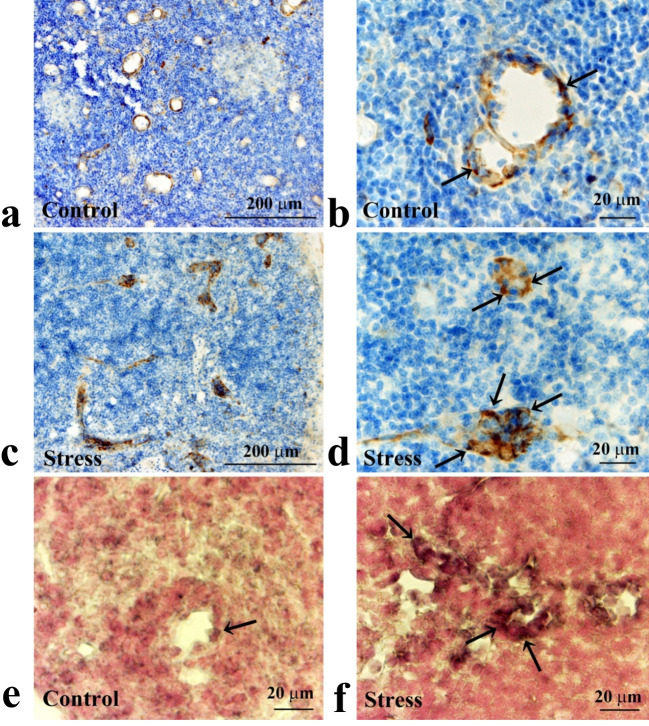
Increases in CXCL2-IR and *Cxcl2*-ISH-positive cells in the 30-min restraint-stressed group. Low (**a**, **c**) and high (**b**, **d**) magnification micrographs of CXCL2-immunoreactive (-IR) endothelial cells of high endothelial venules of control (**a**, **b**) and 30-min restraint-stressed (**c**, **d**) animals. High (**e**, **f**) magnification micrographs of *Cxcl2* in situ hybridization (ISH) histochemical signals from endothelial cells of high endothelial venules of control (**e**) and 30-min restraint-stressed (**f**) animals. Arrows in **b** and **d** indicate CXCL2-IR endothelial cells, and those in **e** and **f** indicate ISH histochemical signals from endothelial cells. Scale bars: **a** and **c** = 200 μm; **b** and **d**–**f** = 20 μm

**Fig. 8:**
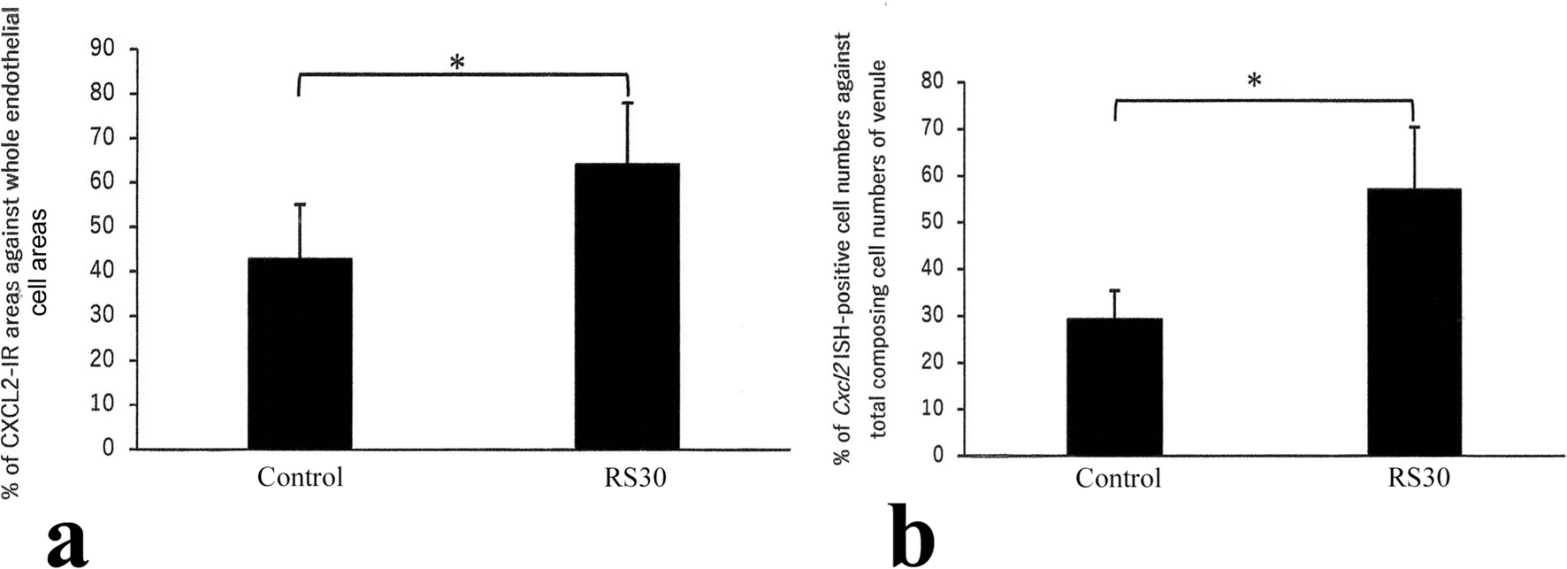
Increases in CXCL2-IR areas and *Cxcl2*-ISH-positive cell numbers in the 30-min restraint-stressed group. Bar graphs indicating the percentage of CXCL2-immunoreactive (-IR) areas compared to whole endothelial cell areas of the high endothelial venules (**a**) and percentage of *Cxcl2* in situ hybridization (ISH)-positive cell number compared to the total number of cells in the high endothelial venules (**b**) in the control and 30-min restraint-stressed (RS30) animals. *P < 0.05 vs. control

Immunohistochemically, CXCL2-immunoreactivity was observed in endothelial cells of the high endothelial venules in the cortex (Fig. 7a–d) but not in those of the ordinal venules in the cortex and medulla. Furthermore, the immunoreactive intensity increased in endothelial cells of the high endothelial venules in the RS30 group (Fig. 7c, d). The percentages of immunopositive areas against the entire endothelial cell area of the high endothelial venules were 42.9 ± 12.2% in the C group and 64.2 ± 13.8% in the RS30 group (*P* < 0.05) (Fig. 8a). Additionally, the ISH findings agreed with those of the immunohistochemical analysis (Fig. 7e, f). Although the positive signals in the cervical lymph nodes were not prominent in the C group (Fig. 7e), they increased significantly in the RS30 group (Fig. 7f). The percentages of *Cxcl2* ISH-positive cell numbers compared to the total number of cells in the high endothelial venules were 29.3 ± 6.1% and 57.2 ± 13.2% in the C and RS30 groups, respectively (*P* < 0.05) (Fig. 8b).

**Fig. 9:**
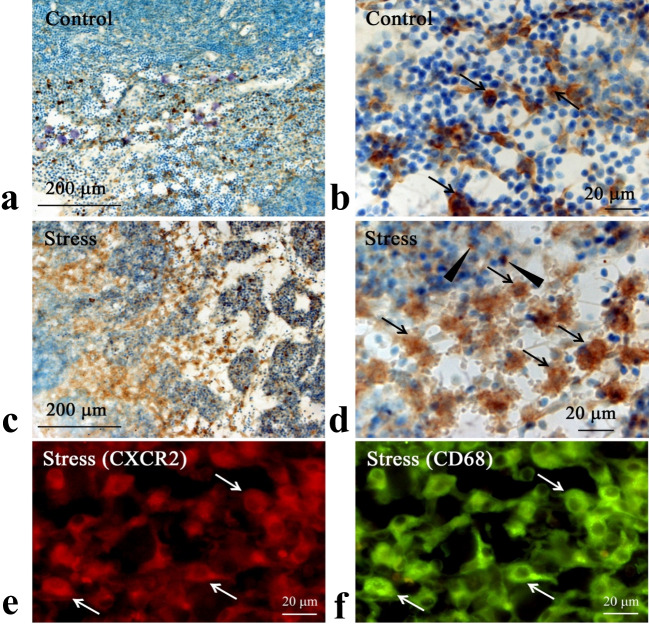
Increases in CXCR2-IR cells in the 30-min restraint-stressed group. Low (**a**, **c**) and high (**b**, **d**) magnification micrographs of CXCR2-immunoreactive (-IR) cells in the medullary sinus of control (**a**, **b**) and 30 min-restraint-stressed (**c**, **d**) animals. Double staining of CXCR2 (**e**) and CD68 (**f**). Arrows in **b** and **d** indicate CXCR2-IR cells, and arrowheads in **d** indicate CXCR2-IR puncta. Arrows in **e** and **f** indicate identical cells. Scale bars: **a** and **c** = 200 μm; **b** and **d**–**f** = 20 μm

**Fig. 10:**
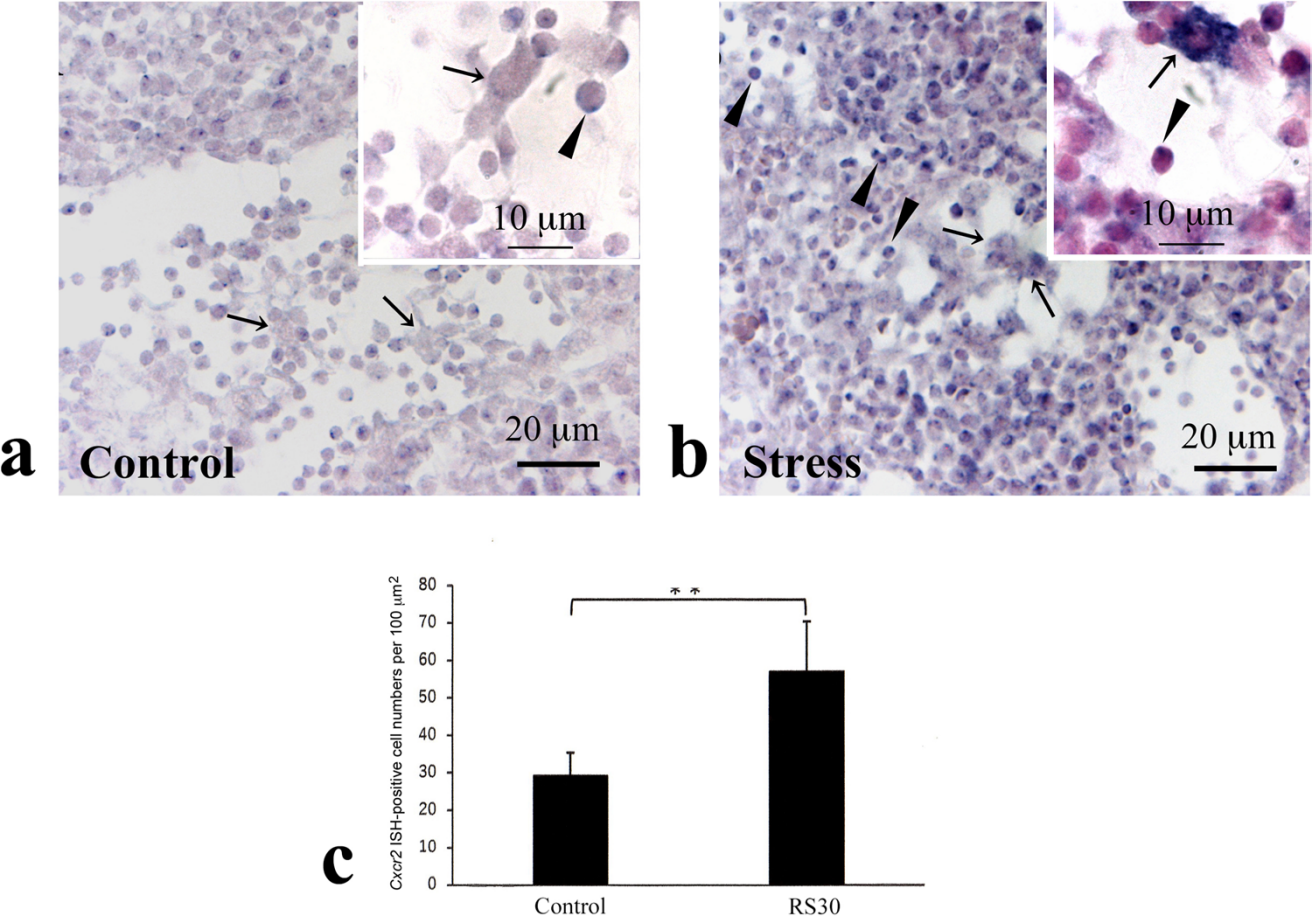
Increases in *Cxcr2*-ISH-positive cells and their cell numbers in the 30-min restraint-stressed group. Low (**a**, **b**) and high (insets) magnification micrographs of *Cxcr2* in situ histochemical signals in medullary sinuses and cords in the control (**a**) and 30-min restraint-stressed (**b**) animals. Arrows in **a**, **b**, and insets indicate presumed macrophages in the medullary sinus. Arrowheads in **a**, **b**, and insets indicate in situ hybridization (ISH)-positive presumed B cells in the medullary sinuses and cords. A bar graph (**c**) indicating *Cxcr2* ISH-positive cell number in the control and 30-min restraint-stressed (RS30) animals per 100 mm^2^. **P < 0.01 vs. control. Scale bars: **a** and **b** = 20 μm; insets = 10 μm

Furthermore, CXCR2-immunoreactive cells were mainly observed in the medullary sinus (Fig. 9a–d). In the C group, the cells were slender and scattered (Fig. 9a, b); in stressed animals, these cells were swollen and denser (Fig. 9c, d). Double staining for CD68 and CXCR2 indicated that CXCR2-immunoreactive cells corresponded to CD68-immunoreactive macrophages (Fig. 9e, f). Furthermore, ISH revealed intense signals in macrophages from the RS30 group (Fig. 10b) compared with those from the C group (Fig. 10a). In addition, *Cxcr*2 signals were observed in the medullary lymphocytes, showing that the intensity was higher in the RS30 group (Fig. 10b) than in the C group (Fig. 10a). The number of *Cxcr2* ISH-positive cells per 100 μm^2^ was 64.7 ± 20.6 and 145.1 ± 34.1 in the C and RS30 groups, respectively (*P* < 0.01) (Fig. 10c).

## Discussion

We demonstrated that RS increased the expression of *Cxcl1*, *Cxcl2*, and *Cxcr2*. The expression of *Cxcl1* and *Cxcl2* induced by RS was transient, whereas that of *Cxcr2* tended to persist for a longer time (Fig. 2). To the best of our knowledge, although no studies have reported on such changes in the cervical lymph nodes, in vivo and in vitro studies have shown that the expression of *Cxcl1*, *Cxcl2*, *and Cxcr2* is synergistically increased at the gene and protein levels upon stimulation in a time-dependent manner [28–31]. These results suggest that changes in expression levels are characteristic biological reactions to RS in the lymph nodes. Continuous expression of *Cxcr2* may correspond to other local environmental changes associated with the expression, networks, and stimulation of other chemokines, such as CXCL3, CXCL4, CXCL5, CXCL6, CXCL7, and CXCL8, because they are also ligands of CXCR2 [32, 33]. Similarly, the expression of *Cxcl3* was increased by 2.32-fold in our study (Fig. 1a). *Cxcl1* was expressed in the medullary cords, where most lymphocytes were B/plasma cells [3]. B/plasma cells are typically activated by invading humoral antigens and produce specific antibodies [3]. However, our results indicate that the B/plasma cells were activated by RS in the absence of invading humoral antigens. Therefore, RS may stimulate B/plasma cells to enhance CXCL1 synthesis and secretion in a pathway distinct from the specific antibody production pathway. Furthermore, *Cxcl2* was expressed in the endothelial cells of the high endothelial venules, suggesting the association of *Cxcl2* with endothelial cell functions (see later discussion). *Cxcr2* was expressed in macrophages and presumable B/plasma cells in the medullary cord. Moreover, the results of GO analysis (Fig. 1b) suggested the involvement of RS-mediated, rapid induction of the CXCL1/CXCL2-CXCR2 axis in leukocyte chemotaxis and migration, as well as in regulating the chemokine network in the cervical lymph nodes and inflammation [34, 35]. We observed that such increases were transient, suggesting that these changes are symptoms of adaptation responses against RS wherein the response is efficiently activated once and then inactivated. Therefore, *Cxcr2*, a receptor for *Cxcl1* and *Cxcl2*, may perform essential roles in concert with *Cxcl1*, *Cxcl2*, and macrophages, to regulate the resistance against RS in the lymph nodes. Although the underlying mechanisms of the adaptation are unknown, some of these effects may be achieved through the nervous system [36, 37] or via inhibitory cytokines [38] (see later discussion). Further studies are required to determine the underlying mechanisms.

Macrophages are heterogeneous with respect to their morphologies, phenotypes, biochemical characteristics, and functions regulated by internal and external factors [39]. Various local growth factors, pro-inflammatory cytokines, and microbial products influence the phenotypic differentiation of macrophages [39, 40]. Macrophages exhibit an array of functions, from phagocytosis and antigen presentation to the production of various cytokines, including interleukin-1, interleukin-6, and tumor necrosis factor α, among others [41, 42]. Stress enhances the infiltration, activation, and antigen-presenting capability of macrophages [15, 43] suggesting that macrophages have a capacity to react against acute stress as demonstrated in this experiment. Although CXCR2 is expressed on macrophage progenitor cells under tumor conditions [44], to the best of our knowledge, this is the first study to demonstrate that CXCR2 is expressed on macrophages under non-tumor conditions. Interestingly, CXCL1 and CXCL2, which were both increased by RS, are ligands of the CXCR2 receptor. Stress-induced CXCL1 and CXCL2 may activate macrophages via CXCR2 and result in erythrocyte accumulation seen in the RS30. Taken together, a CXCL1/CXCL2 and CXCR2 axis may perform key functions in the cervical lymph nodes in response to acute stress.

We demonstrated that acute stress increased CXCL2 in high endothelial venules of the cervical lymph nodes at the transcript and protein levels. Regarding high endothelial venules, lymphocytes migrate from the bloodstream to the lymph node matrix [45–48]. The recruitment of leukocytes, including lymphocytes, is precisely regulated by selective leukocyte-endothelial cell recognition and involves at least three consecutive steps: primary adhesion, chemoattractant activation, and activation-dependent adhesion [46, 47]. Each step requires various factors and adhesion receptors and several combinatorial mechanisms to generate specificity and diversity in leukocyte-endothelial cell interactions. Among these three steps, the second step involves multiple chemokines, including those of the CXC and CC families [46, 47]. Although CXCL1 is defined as an endothelial-derived chemoattractant, CXCL2 is not listed under this category. However, Springer [47] proposed that other chemokines are excellent candidates for providing the signal (second step) required to activate integrin adhesiveness and lymphocyte migration to the lymph nodes. In support of this proposal, our findings suggest that CXCL2 is an acute stress-related endothelial-derived chemoattractant in the lymph nodes. Therefore, the rapid stress-induced CXCL2 and CXCL1 play important roles in leukocyte chemotaxis and migration within the cervical lymph nodes to achieve immune responses against acute stress.

Acute stress activates the sympathetic nervous system and hypothalamus–pituitary–adrenal axis, resulting in a rapid increase in the levels of plasma stress hormones, including catecholamines and glucocorticoids [20, 21]. Innervation of the lymph nodes by sympathetic nerve fibers is well-documented [49, 50], where noradrenergic fibers penetrate along with blood vessels from the lymph node hilus to the cortical regions. The fibers extend from the blood vessel nerve plexus to regions of T lymphocytes in the paracortical and cortical zones [49]. Adrenergic receptors (AR) were previously detected in immune cells [51]. Among the ARs, βAR is associated with cells responsible for innate immunity, and αAR is primarily expressed by monocytes/macrophages [50]. Moreover, the intimate physiological interaction between nerve fibers and immune cells was morphologically observed in the lymph nodes [52]. Activation of ARs leads to the production of inflammatory substances such as cytokines [53]. Other prominent stress hormones, such as glucocorticoids, exert anti-inflammatory and immuno-suppressive properties by regulating immune cell adhesion on blood vessel endothelial cells, suppressing immune cell activation, and inhibiting cytokine production [19, 54]. Furthermore, glucocorticoids downregulate pro-inflammatory mediators from monocytes/macrophages and differentiate into anti-inflammatory phenotypes, ultimately increasing migration [55]. Collectively, although the underlying mechanisms remain unclear, increased levels of catecholamines and glucocorticoids induced by acute stress may directly or indirectly be associated with the transient activation of macrophages and the CXCL1/CXCL2-CXCR2 axis in the cervical lymph node microenvironment.

The present study was performed using animal models. Although the results obtained in animal models are not directly applicable to humans, many types of these models have been established [56, 57], and data that reflect human health have been obtained. The most well-known case is the “stress theory” proposed in 1936 by Hans Selye, who put forward the theory based on data from animal models [58]. Since then, our understanding of human stress has considerably improved, and some insights have been applied in clinical settings. Therefore, our data provide a foundation for further studies in humans.

## Data Availability

All data generated or analyzed during this study are included in this published article. Microarray data has been registered with GEO (GSE202994: https://www.ncbi.nlm.nih.gov/geo/query/acc.cgi?acc=GSE202994).

## References

[1] Barker CF, Billingham RE (1968). The role of afferent lymphatics in the rejection of skin homografts. J Exp Med..

[2] Gretz JE, Anderson AO, Shaw SS (1997). Cords, channels, corridors and conduits: critical architectural elements facilitating cell interactions in the lymph node cortex. Immunol Rev..

[3] Willard-Mack CL (2006). Normal structure, function, and histology of lymph nodes. Toxicol Pathol..

[4] Ohtani O, Ohtani Y (2008). Structure and function of rat lymph nodes. Arch Histol Cytol..

[5] von Andrian UH, Mempel TR (2003). Homing and cellular traffic in lymph nodes. Nat Rev Immunol..

[6] Schilling C, Stoeckli SJ, Haerle SK, Broglie MA, Huber GF, Sorensen JA (2015). Sentinel European Node Trial (SENT): 3-year results of sentinel node biopsy in oral cancer. Eur J Cancer..

[7] Gerard C, Rollins BJ (2001). Chemokines and disease. Nat Immunol..

[8] Zlotnik A, Yoshie O, Nomiyama H (2006). The chemokine and chemokine receptor superfamilies and their molecular evolution. Genome Biol..

[9] Rossi D, Zlotnik A (2000). The biology of chemokines and their receptors. Annu Rev Immunol..

[10] Liu X, Dai LI, Zhou R (2015). Association between preeclampsia and the CXC chemokine family (Review). Exp Ther Med..

[11] Lysle DT, Lyte M, Fowler H, Rabin BS (1987). Shock-induced modulation of lymphocyte reactivity: suppression, habituation, and recovery. Life Sci..

[12] Aarstad HJ, Kolset SO, Seljelid R (1991). The effect of stress in vivo on the function of mouse macrophages in vitro. Scand J Immunol..

[13] Durant S, Coulaud J, Amrani A, el Hasnaoui A, Dardenne M, Homo-Delarche F (1993). Effects of various environmental stress paradigms and adrenalectomy on the expression of autoimmune type 1 diabetes in the non-obese diabetic (NOD) mouse. J Autoimmun..

[14] Dhabhar FS, McEwen BS (1997). Acute stress enhances while chronic stress suppresses cell-mediated immunity *in vivo*: a potential role for leukocyte trafficking. Brain Behav Immun..

[15] Viswanathan K, Daugherty C, Dhabhar FS (2005). Stress as an endogenous adjuvant: augmentation of the immunization phase of cell-mediated immunity. Int Immunol..

[16] Sasaguri K, Yamada K, Yamamoto T (2018). Uncovering the neural circuitry involved in the stress-attenuation effects of chewing. Jpn Dent Sci Rev..

[17] Onuki M, Yamamoto T, Sasaguri K, Yamada K, Okada N, Kawata T (2018). Chewing ameliorates the effects of restraint stress on pERK-immunoreactive neurons in the rat insular cortex. Neurosci Lett..

[18] Hatanaka R, Onuki M, Sasaguri K, Yamada K, Saruta J, Yamamoto T (2020). Chewing augments stress-induced increase of pERK-immunoreactive cells in the rat cingulate cortex. Neurosci Lett..

[19] Chrousos GP (1995). The hypothalamic-pituitary-adrenal axis and immune-mediated inflammation. N Engl J Med..

[20] Webster Marketon JIW, Glaser R (2008). Stress hormones and immune function. Cell Immunol..

[21] Webster JI, Tonelli L, Sternberg EM (2002). Neuroendocrine regulation of immunity. Annu Rev Immunol..

[22] Sasaguri K, Yamada K, Narimatsu Y, Oonuki M, Oishi A, Koda K (2017). Stress-induced galectin-1 influences immune tolerance in the spleen and thymus by modulating CD45 immunoreactive lymphocytes. J Physiol Sci..

[23] Tsai CY, Fang C, Wu JCC, Wu CJ, Dai KY, Chen SM (2021). Neuroinflammation and microglial activation at rostral ventrolateral medulla underpin cadmium-induced cardiovascular dysregulation in rats. J Inflam Res..

[24] Piotrowska A, Rojewska E, Pawlik K, Kreiner G, Ciechanowska A, Makuch W (2019). Pharmacological blockade of spinal CXCL3/CXCR2 signaling by NVP CXCR2 20, a selective CXCR2 antagonist, reduces neuropathic pain following peripheral nerve injury. Front Immunol..

[25] Chi ZL, Adini A, Birsner AE, Bazinet L, Akula JD, D’Amato RJ (2019). PR1P ameliorates neurodegeneration through activation of VEGF signaling pathway and remodeling of the extracellular environment. Neuropharmacology..

[26] Kaneko T, Myo Zaw SY, Sueyama Y, Katsube KI, Kaneko R, Nör JE (2019). Inhibition of nuclear factor kappa B prevents the development of experimental periapical lesions. J Endod..

[27] Sougawa N, Miyagawa S, Kawamura T, Matsuura R, Harada A, Sakai Y (2021). Combined administration of laminin-221 and prostacyclin agonist enhances endogenous cardiac repair in an acute infarct rat heart. Sci Rep..

[28] Yellowhair TR, Noor S, Maxwell JR, Anstine CV, Oppong AY, Robinson S (2018). Preclinical chorioamnionitis dysregulates CXCL1/CXCR2 signaling throughout the placental-fetal-brain axis. Exp Neurol..

[29] Chen MC, Baskaran R, Lee NH, Hsu HH, Ho TJ, Tu CC (2019). CXCL2/CXCR2 axis induces cancer stem cell characteristics in CPT-11-resistant LoVo colon cancer cells via Gαi-2 and Gαq/11. J Cell Physiol..

[30] Chen F, Wang D, Li X, Wang H (2020). Molecular mechanisms underlying intestinal ischemia/reperfusion injury: bioinformatics analysis and in vivo validation. Med Sci Monit..

[31] Ni H, Wang Y, An K, Liu Q, Xu L, Zhu C (2019). Crosstalk between NFκB-dependent astrocytic CXCL1 and neuron CXCR2 plays a role in descending pain facilitation. J Neuroinflammation..

[32] Yamamoto Y, Kuroda K, Sera T, Sugimoto A, Kushiyama S, Nishimura S (2019). The clinicopathological significance of the CXCR2 ligands, CXCL1, CXCL2, CXCL3, CXCL5, CXCL6, CXCL7, and CXCL8 in gastric cancer. Anticancer Res..

[33] Korbecki J, Kojder K, Kapczuk P, Kupnicka P, Gawrońska-Szklarz B, Gutowska I (2021). The effect of hypoxia on the expression of CXC chemokines and CXC chemokine receptors-a review of literature. Int J Mol Sci..

[34] Zhou H, Lei PJ, Padera TP (2021). Progression of metastasis through lymphatic system. Cells..

[35] Louie DAP, Liao S (2019). Lymph node subcapsular sinus macrophages as the frontline of lymphatic immune defense. Front Immunol..

[36] Sousa N, Almeida OF (2012). Disconnection and reconnection: the morphological basis of (mal) adaptation to stress. Trends Neurosci..

[37] Wu C, Sartor RB, Huang K, Tonkonogy SL (2016). Transient activation of mucosal effector immune responses by resident intestinal bacteria in normal hosts is regulated by interleukin-10 signalling. Immunology..

[38] Bonini D, Mora C, Tornese P (2016). Acute footshock induces time-dependent modifications of AMPA/NMDA protein expression and AMPA phosphorylation. Neural Plast..

[39] Epelman S, Lavine KJ, Randolph GJ (2014). Origin and functions of tissue macrophages. Immunity..

[40] Nourshargh S, Alon R (2014). Leukocyte migration into inflamed tissues. Immunity..

[41] Sica A, Erreni M, Allavena P, Porta C (2015). Macrophage polarization in pathology. Cell Mol Life Sci..

[42] Wynn TA, Chawla A, Pollard JW (2013). Macrophage biology in development, homeostasis and disease. Nature..

[43] Knop J, Malorny U, Michels E, Sorg C (1984). Selection of the delayed hypersensitivity T effector and T suppressor cell response by antigen-presenting macrophages. Immunobiology..

[44] Han X, Shi H, Sun Y, Shang C, Luan T, Wang D (2019). CXCR2 expression on granulocyte and macrophage progenitors under tumor conditions contributes to mo-MDSC generation via SAP18/ERK/STAT3. Cell Death Dis..

[45] Grizzi F, Borroni EM, Vacchini A, Qehajaj D, Liguori M, Stifter S (2015). Pituitary adenoma and the chemokine network: a systemic view. Front Endocrinol (Lausanne)..

[46] Butcher EC (1991). Leukocyte-endothelial cell recognition: three (or more) steps to specificity and diversity. Cell..

[47] Springer TA (1994). Traffic signals for lymphocyte recirculation and leukocyte emigration: the multistep paradigm. Cell..

[48] Salmi M, Jalkanen S (1997). How do lymphocytes know where to go: current concepts and enigmas of lymphocyte homing. Adv Immunol..

[49] Felten DL, Felten SY, Carlson SL, Olschowka JA, Livnat S (1985). Noradrenergic and peptidergic innervation of lymphoid tissue. J Immunol..

[50] Nance DM, Sanders VM (2007). Autonomic innervation and regulation of the immune system (1987–2007). Brain Behav Immun..

[51] Kin NW, Sanders VM (2006). It takes nerve to tell T and B cells what to do. J Leukoc Biol..

[52] Hu D, Nicholls PK, Claus M, Wu Y, Shi Z, Greene WK (2019). Immunofluorescence characterization of innervation and nerve-immune cell interactions in mouse lymph nodes. Eur J Histochem..

[53] Ordovas-Montanes J, Rakoff-Nahoum S, Huang S, Riol-Blanco L, Barreiro O, von Andrian UH (2015). The regulation of immunological processes by peripheral neurons in homeostasis and disease. Trends Immunol..

[54] Goulding NJ, Ogbourn S, Pipitone N, Biagini P, Gerli R, Pitzalis C (1999). The inhibitory effect of dexamethasone on lymphocyte adhesion molecule expression and intercellular aggregation is not mediated by lipocortin 1. Clin Exp Immunol..

[55] Ehrchen JM, Roth J, Barczyk-Kahlert K (2019). More than suppression: glucocorticoid action on monocytes and macrophages. Front Immunol..

[56] Sutanto W, de Kloet ER (1994). The use of various animal models in the study of stress and stress-related phenomena. Lab Anim..

[57] Carnevali L, Montano N, Tobaldini E, Thayer JF, Sgoifo A (2020). The contagion of social defeat stress: insights from rodent studies. Neurosci Biobehav Rev..

[58] Szabo S, Yoshida M, Filakovszky J, Juhasz G (2017). ‘Stress’ is 80 years old: from Hans Selye original paper in 1936 to recent advances in giulceration. Curr Pharm Des..

